# An Inflammatory Bowel Diseases Integrated Resources Portal (IBDIRP)

**DOI:** 10.1093/database/baad097

**Published:** 2024-01-16

**Authors:** Nie Kai, Cai Qingsong, Ma Kejia, Luo Weiwei, Wu Xing, Chen Xuejie, Cai Lixia, Deng Minzi, Yang Yuanyuan, Wang Xiaoyan

**Affiliations:** Department of Gastroenterology, The Third Xiangya Hospital of Central South University, Changsha Hunan 410000, China; Hunan Key Laboratory of Nonresolving Inflammation and Cancer, Changsha Hunan 410000, China; Department of Gastroenterology, The Third Xiangya Hospital of Central South University, Changsha Hunan 410000, China; Hunan Key Laboratory of Nonresolving Inflammation and Cancer, Changsha Hunan 410000, China; Department of Gastroenterology, The Third Xiangya Hospital of Central South University, Changsha Hunan 410000, China; Hunan Key Laboratory of Nonresolving Inflammation and Cancer, Changsha Hunan 410000, China; Department of Gastroenterology, The Third Xiangya Hospital of Central South University, Changsha Hunan 410000, China; Hunan Key Laboratory of Nonresolving Inflammation and Cancer, Changsha Hunan 410000, China; Department of Gastroenterology, The Third Xiangya Hospital of Central South University, Changsha Hunan 410000, China; Hunan Key Laboratory of Nonresolving Inflammation and Cancer, Changsha Hunan 410000, China; Changsha Hospital for Maternal and Child Health Care Affiliated to Hunan Normal University Changsha Hunan 410000, China; Department of Gastroenterology, The Third Xiangya Hospital of Central South University, Changsha Hunan 410000, China; Hunan Key Laboratory of Nonresolving Inflammation and Cancer, Changsha Hunan 410000, China; Department of Gastroenterology, The Third Xiangya Hospital of Central South University, Changsha Hunan 410000, China; Hunan Key Laboratory of Nonresolving Inflammation and Cancer, Changsha Hunan 410000, China; Department of Gastroenterology, The Third Xiangya Hospital of Central South University, Changsha Hunan 410000, China; Hunan Key Laboratory of Nonresolving Inflammation and Cancer, Changsha Hunan 410000, China; The College of Computer Science in Sichuan University, Chengdu Sichuan 610000, China

## Abstract

IBD, including ulcerative colitis and Crohn’s disease, is a chronic and debilitating gastrointestinal disorder that affects millions of people worldwide. Research on IBD has generated massive amounts of data, including literature, metagenomics, metabolomics, bioresources and databases. We aim to create an IBD Integrated Resources Portal (IBDIRP) that provides the most comprehensive resources for IBD. An integrated platform was developed that provides information on different aspects of IBD research resources, such as single-nucleotide polymorphisms (SNPs), genes, transcriptome, microbiota, metabolomics, single cells and other resources. Valuable and comprehensive IBD-related data were collected from PubMed, Google, GMrepo, gutMega, gutMDisorder, Single Cell Portal and other sources. Then, the data were systematically sorted, and these resources were manually curated. We systematically sorted and cataloged more than 320 unique risk SNPs associated with IBD in the SNP section. We presented over 289 IBD-related genes based on the database collection in the gene section. We also obtained 153 manually curated IBD transcriptomics data, including 12 388 samples, on the Gene Expression Omnibus database. The sorted IBD-related microbiota data from three primary microbiome databases (GMrepo, gutMega and gutMDisorder) were available for download. We selected 23 149 IBD-related taxonomic records from these databases. Additionally, we collected 24 IBD metabolomics studies with 2896 participants in the metabolomics section. We introduced two interactive single-cell data plug-in units that provided data visualization based on cells and genes. Finally, we listed 18 significant IBD web resources, such as the official European Crohn’s and Colitis Organisation and International Organization for the Study of IBD websites, IBD scoring tools, IBD genetic and multi-omics resources, IBD biobanks and other useful research resources. The IBDIRP website is the first integrated resource for global IBD researchers. This portal will help researchers by providing comprehensive knowledge and enabling them to reinforce the multidimensional impression of IBD. The IBDIRP website is accessible via www.ibdirp.com

**Database URL**: www.ibdirp.com.

## Introduction

IBDs, represented by Crohn’s disease (CD) and ulcerative colitis (UC), are chronic idiopathic inflammatory conditions of the gastrointestinal tract. UC is mostly restricted to the colon with confluent mucosal inflammation, whereas CD manifests as transmural inflammation in bowel segments, most often ileocecum (1). IBD incidence and prevalence have been increasing worldwide, with different frequencies depending on ethnicity and geography (2). IBDs affect the quality of life due to diarrhea, abdominal pain, bloody stool, fatigue and weight loss (1). Patients require long-term anti-inflammatory, immunosuppressive, immunomodulatory medications and surgery to control disease activity because the disease is remitting and recurring (3), resulting in a substantial burden to patients and healthcare systems.

The mechanism of IBD pathogenesis is multifactorial, involving intricate interactions between genes, the environment, microbiota and potentially other factors, leading to dysregulation of the immune system (4–6). Genome-wide association studies (GWAS) sequencing the whole genome of large case–control cohorts to identify genes and single-nucleotide polymorphisms (SNPs) associated with a particular disease or another trait. GWAS has identified more than 300 risk loci associated with IBD in different populations (7–10).

However, most genetic variants have a relatively minor effect on disease risk, and non-genetic factors, such as the environment and intestinal microbiota, also modulate this effect (11, 12). Firmicutes, Bacteroidetes, Proteobacteria and Actinobacteria comprise 99% of the intestinal microbiota in healthy individuals and perform vital functions such as nourishment and immune regulation via host–microbe interactions (13). Patients with IBD have reduced microbial diversity and relative abundance of Firmicutes while increasing Proteobacteria and Bacteroidetes (14–16). Microbial metabolic activity is important for resisting intestinal inflammation, and microbiota alterations can potentially contribute to the inflammatory state of IBD. For example, short-chain fatty acids produced by Firmicutes and Bacteroidetes play crucial roles in protecting the intestinal epithelium (17).

Single-cell RNA sequencing (scRNA-seq) has significantly improved over the past 5 years. This technology provided unprecedented insights into refining cell-type signatures, uncovering cellular heterogeneity and suggesting new cell types (18). In the past decade, large-scale omics data have unraveled the pathogenesis of IBD, including the literature, SNP data, metagenomics, metabolomics, single-cell sequencing data, bioresources and databases.

Although data from numerous studies have provided enormous information regarding IBD, they can be incomprehensive and conflicting (19), necessitating data integration using different omics approaches. Researchers have developed some online resources on narrow topics during the early stages to facilitate understanding of IBD. These databases included UC Database (UCDB) (20), IBD Database (IBDDB) (21) and IBD Multi-omics Database (IBDMDB) (22). The rapid advancement of new technologies, such as next-generation sequencing, 10X genomics and multi-omics, has resulted in rapid data accumulation and potential exploration in IBD research. In this study, we tried this direction and elaborated an IBD Integrated Resources Portal (IBDIRP) with the most comprehensive resources, including SNP, gene, microbiota, metabolomics, single cell and other resources. Overall, IBDIRP is a specialized, first integrated resource for IBD researchers that enables the exploration of multidimensional sequence and experimental data.

## Materials and Methods

### Data collection and website building

‘FanKe’ (https://www.fkw.com/) was used as a software-as-a-service–based online web design service. The website was built using Java Server Pages (JSP), and an adaptive template was created using JSP and Node.js technologies. The website can realize the adaptation of computers and mobile phones, and different terminals can obtain the same presentation effect. The IBDIRP website was saved in a MySQL-based business database, and the operating system was Linux CentOS7.0. Several independent sections were built based on our collected IBD-related research resources, including the home page, SNP section, gene section, transcriptomics section, microbiota section, single-cell section, metabolomics section, other resources section and our lab section. Webpage optimization was also introduced in the display of complex research resources. Several website interactivities were developed, including picture-click-jump outer website, interactive web plug-in and download resources.

### Single-nucleotide polymorphism

Multiple significant studies on SNPs within the PubMed database underwent comprehensive review. The process involved meticulous manual curation of primary SNP data associated with IBD sourced from literature. This manual extraction of information was deemed valuable by the authors as it assured the database’s credibility and reliability. This ongoing curation method was periodically conducted to incorporate the latest findings in the field. Several crucial factors, including the inclusivity of SNP studies, journal credibility, utilization of meta-analyses, result significance and representativeness, were meticulously evaluated during the selection process.

The most comprehensive and latest meta-IBD SNP data have been listed in this section. Since these data typically originate from the European and American sources, other regional IBD SNP data, such as Asian data, have also been considered to provide a representative perspective in this section.

### Genes

PubMed, Web of Science and other databases were searched for IBD-related gene studies. The primary data on genes involved in IBD were collected manually from the literature, in which the authors’ opinion was an added value of the implemented database because it guaranteed high-resource reliability. The data collected by other groups from similar databases, systemic reviews and online knowledge tools on Google, Twitter and others were also considered. The information embedded in concepts was acquired via text mining of PubMed (manually cleaned and curated), accompanied by data mining from varied resources. The detailed method was introduced previously by Khan *et al*. (21). The representative data that could reflect IBD-related pathophysiology were chosen for convenience and were manually updated.

### Transcriptome

RNAseq technologies, such as Sanger, Illumina, Pacbio and Nanopore, are available to study RNA biology, including gene expression and translation (the translatome) (23). This contributes to a better understanding of RNA biology (23). Numerous transcriptomics of IBD were stored in the Gene Expression Omnibus (GEO) (24), National Genomics Data Center (25), European Bioinformatics Institute (EBI) (26) and other databases. The GEO database is the largest transcriptome database, and the search qualifications were (IBD OR Crohn’s OR Ulcerative colitis [Title], Homo sapiens [Organism], expression by array and high-throughput sequencing [Study type]). IBD transcriptomics data under several search qualifications in the GEO were listed in the transcriptomic section. User also could supplement the data by clicking the other database icon.

### Microbiota

We thoroughly searched the microbiome databases from three independent source: Google, PubMed and *Nucleic Acids Research* Database Summary (https://www.oxfordjournals.org/nar/database/subcat/8/34). Three reported integrated gut microbiota databases were found: GMrepo (27), gutMega (28) and gutMDisorder (29). These data provided unbiased information for IBD researchers. GMrepo is reported the most integrated gut microbiota database. We gave a brief curated IBD dysbiosis map derived from the GMrepo database (27). A total of 23 149 taxonomic records were selected from these three databases, and the sorted IBD-related microbiota data from three primary independent microbiome databases (GMrepo, gutMega and gutMDisorder) were available for download.

### Metabolomics

Large IBD metabolomics studies in PubMed were searched and sorted for important data, including detailed IBD phenotypes, good quality control, large sample sizes and global representativeness. We collected all the recently published large-scale IBD metabolomics studies from the PubMed database. Each study listed the author, specific IBD phenotypes, study title, metabolomics type, number of volunteers, nation, control information and PubMed Unique Identifier (PMID) number. A manually curated table was built to display IBD metabolomics data.

### Single cells

IBD single-cell data were searched on major IBD single-cell data storage platforms, including Single Cell Portal (SCP, https://singlecell.broadinstitute.org/single_cell), scIBD database (http://scibd.cn), Single Cell Expression Atlas (https://www.ebi.ac.uk/gxa/sc/home), PanglaoDB (https://panglaodb.se/index.html), GEO (https://www.ncbi.nlm.nih.gov/geo), Human Cell Atlas (HCA, https://www.humancellatlas.org/), Cell-omics Data Coordinate Platform (https://db.cngb.org/cdcp/), scRNASeqDB (https://bioinfo.uth.edu/scrnaseqdb/), SCPortalen (http://single-cell.clst.riken.jp/), SC2disease (http://easybioai.com/sc2disease) and PubMed (https://pubmed.ncbi.nlm.nih.gov/). To better visualize IBD cell-specific signature based on single-cell data, two typical IBD subtypes (UC and CD) single-cell plug-in interactive webpages were sorted to visualize the IBD single-cell data.

### Other resources

Multiple IBD website resources were sorted from Google, PubMed, Baidu and Twitter. The priority was given to resources that can promote the accessibility of IBD knowledge and facilitation of IBD researchers, including IBD professional organizations, IBD management tools, IBD single-item databases and IBD biological research resources. Representative and strong resources were selected from our other resources section. Simultaneously, a brief IBD description webpage and our lab introduction page were built to make the resources portal more readable and integral.

## Results

**Figure 1.**
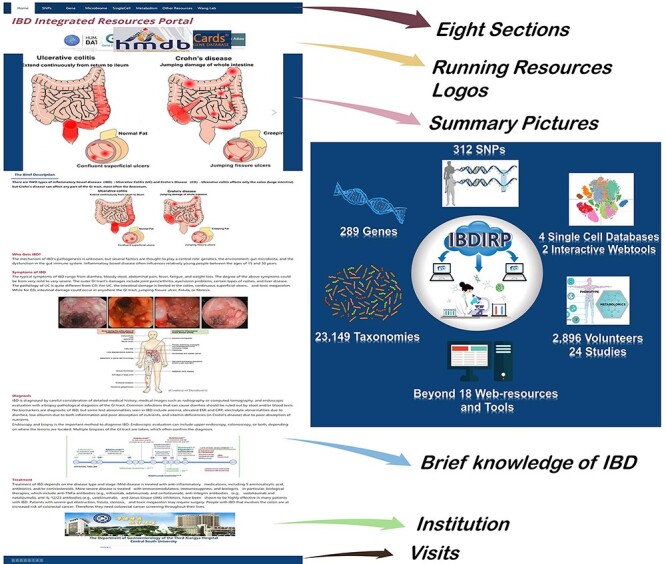
The homepage’s introduction and the overview of IBDIRP website. IBDIRP sorted and cataloged more than 320 unique risk SNPs associated with IBD, 289 IBD-related genes, 153 IBD transcriptomics data, including 12 388 samples, 23 149 IBD-related taxonomic records, 24 IBD metabolomics studies with 2896 participants, 18 significant IBD web resources and other useful research resources.

We built an IBDIRP based on multiple results of our extensive search. IBD resources include but are not limited to PubMed, Web of Science, Google, microbiota databases, single-cell databases and other professional databases, as well as social media content, such as Twitter. We intend not to build a full, large, bloated and slow resource storage station but to build a multifaceted query platform that can respond quickly and facilitate research queries anytime and anywhere. Simultaneously, we attempt to reinforce the integrated impression of multiple pathophysiological views of IBD and emphasize the connection and close interaction between genetics, microbiota, metabolites and other omics data. Seven sections display these abundant IBD research resources, including SNPs, genes, transcriptomics, microbiota, metabolomics, single cells and other resources (Figure 1). Researchers can enhance their research projects by integrating multiple section data to identify core components, for instance, identifying core IBD-associated SNPs, key IBD genes, important microbial communities specific to IBD, essential metabolites and core cell types. The compilation of these sections can contribute to the construction of a comprehensive macro-modulation map for IBD. The cross mapping, interaction and overlapping intersection based on the multidimensional data we provide can better support the IBD researcher’s own work. Developing new methodology to reuse our collective data will greatly reduce the repeated sampling, sequencing and resource-wasting. In addition to displaying brief integrated IBD research resources and download resources, we provide numerous interactivities based on the ‘click to jump outside’ mode, encouraging visitors to investigate more detailed research resources. Two plug-in web pages encouraged visitors to explore more interactive results.

**Alt Text**: The homepage’s introduction and the brief overview of IBDIRP website.

### SNP section

**Figure 2.**
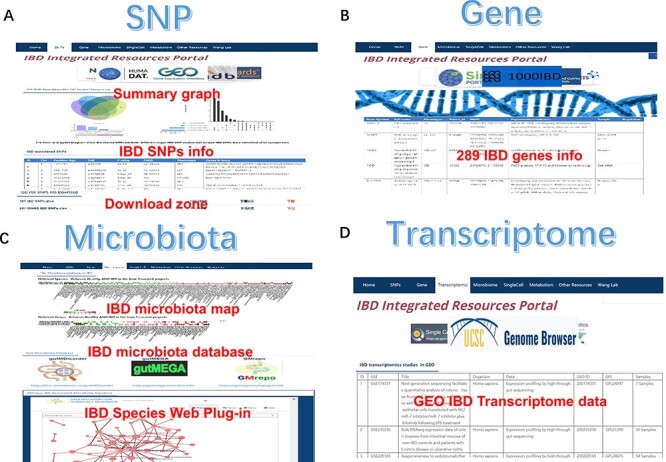
The SNP, gene, microbiota and transcriptome section of IBDIRP. (**A**) The SNP section conllected more than 320 unique risk SNPs associated with IBD. (**B**) The gene section displays 289 IBD-related genes based on the database’s collection. (**C**) The sorted IBD-related microbiota data are available in microbiota section. (**D**) The transcriptome section displays 153 IBD-related transcriptome data.

GWAS have identified many risk variants (NOD2, ATG16L1, IL23R and IL10) (6). Disease-associated SNPs usually influence disease states; many IBD-associated SNPs have been detected using GWAS and other technologies (8, 30). These variants could influence RNA fate and gene expression by affecting RNA modifications. Many IBD variants detected so far have not been well explained or fine-mapped, and their related genes commonly used to describe them are only putative. Moreover, the biological functions of their products and their complex interactions require delineation in most cases. We sorted over 320 unique risk SNPs associated with IBD in the SNP section (Figure 2). These SNPs primarily originate from four large IBD SNP metastudies, including 75 000 cases and controls (31), 86 640 Europeans and 9846 Asians (7), 25 305 European IBD cases and controls (8) and Asians (32). The UpSet diagram exhibited shared and unshared SNP in these four studies. We displayed the results of these four studies’ independently, including 241 SNPs (8), 167 SNPs (31), 38 SNPs (7) and 17 SNPs (32) due to different populations and criteria. Liu *et al*. reported the largest IBD risk SNP among East Asian ancestries, including 14 393 cases and 15 456 controls (33). They discovered 320 IBD loci when meta-analyzed with ∼370 000 Europe individuals (∼30 000 cases). This new GWAS result has also been included in our SNP section. The download resources are provided at the bottom of this section.

**Alt Text**: The brief introduction of SNP, gene, microbiota and transcriptome section in IBDIRP website.

### Gene section

IBDDB manually curates 289 genes that have been experimentally validated to be linked to IBD and its known phenotypes (21). Furthermore, it provides information regarding different aspects of these genes by incorporating several resources and an extensive text-mining knowledge base. It allows the selective display of collated 34 subject-specific concepts (listed as columns) exportable via a user-friendly IBDDB portal (21). The information embedded in concepts was acquired via text mining of PubMed (manually cleaned and curated), accompanied by data mining from various resources. We displayed over 289 studied IBD-related genes based on the database’s collection from IBDDB data in the gene section (Figure 2). The online gene list provides detailed gene symbols, gene full name, IBD phenotypes, entrez_id, PMID, experimental method evidence, sample and regulation. A download resource is also available to the researchers in this section.

### Transcriptomics section

**Figure 3.**
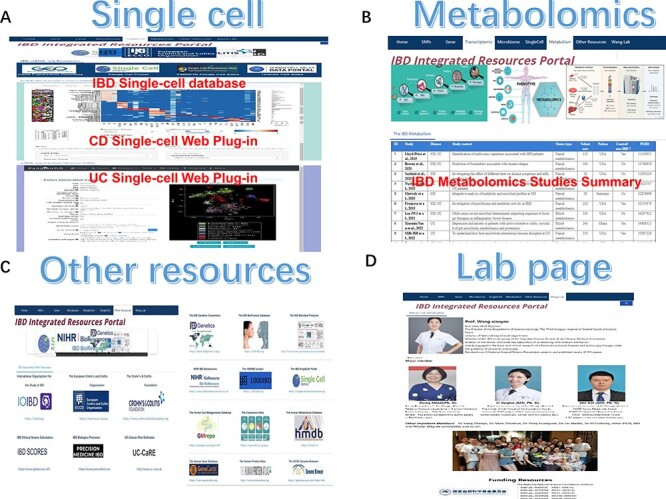
The single-cell, metabolomics, other resources and lab page section of IBDIRP. (**A**) The single-cell section exhibited major IBD-related database and introduced IBD single-cell interactive visualization. (**B**) More than 24 primary recently published large-scale IBD metabolomics studies are displayed in the metabolomics section. (**C**) The other research resource listed 18 important IBD web resources. (**D**) The lab page introduces our lab briefly.

We developed a manually curated table to display the research results of IBD-related transcriptomics data under conditional search qualifications in the GEO database (24). A total of 153 IBD-related expression data contain five datasets, 153 series and 12 388 samples to date (Figure 3). The web table provides the title, organism, data type, platform, accession ID and sample number of every study. The GEO IBD data summary, including descriptions and links, was uploaded to the download zone.

**Alt Text**: The brief introduction of single-cell, metabolomics, other resources and lab page section in IBDIRP website.

### Microbiota section

Despite IBD-related genetic resources, microbiota dysbiosis is a significant characteristic of IBD and influences its pathophysiology, mostly from the immune, metabolic and gut barrier systems. The integrated meta-analysis data may be better for understanding the changes in IBD gut microbiota because IBD microbiota studies vary in samples, populations, methods and medications. We provide a brief curated IBD dysbiosis map derived from the GMrepo database, which is a comprehensive gut microbiome database (27) (Figure 2). Three reported integrated gut microbiota databases were GMrepo (https://gmrepo.humangut.info) (27), gutMega (http://gutmega.omicsbio.info) (28) and gutMDisorder (http://bio-annotation.cn/gutMDisorder) (29), which offer us channels to catch a gut microbiota dysbiosis glimpse from massive integrated metagenome data. A total of 23 149 taxonomic records were selected from these databases, and the sorted IBD-related microbiota data from three primary independent microbiome databases (GMrepo, gutMega and gutMDisorder) were available for download (Figure 2).

### Metabolomics section

Metabolomics is the frontline of IBD-associated multi-omics studies; data from IBD metabolomics enrich our understanding of the close and complex interaction mechanisms among diet, microorganisms and intestinal cells. In particular, detailed gut organs, such as the gut–brain axis, gut–liver axis and gut–adipose axis, rely on signals from gut metabolites, and genes and cells interact with metabolites tightly. Large-scale IBD metabolomics studies are still limited and have recently been on the frontline of IBD research. We collected 24 primary recently published large-scale IBD metabolomics studies from the metabolomics section (Figure 3). Each study listed the author, specific IBD phenotypes, study title, metabolomics type, number of volunteers, nation, control information and PMID number. These data may provide an initial guide for IBD metabolic research.

### Single-cell section

Single-cell data are complex and huge, but it is a good way to understand specific detailed subtle shifts and functional changes in cell subsets. Single-cell sequencing of IBD is the most innovative and remarkable technological advancement, precisely linking microbes, metabolites, cells and genes. Recently, a comprehensively integrated analysis platform for IBD scRNA-seq datasets was developed. scIBD, a platform for single-cell meta-analysis of IBD, combines highly curated single-cell datasets, enabling identifying rare or less-characterized cell types in IBD and dissecting the commonalities and differences between UC and CD (34). We listed five primary IBD-related single-cell data storage platforms: GEO (24), SCP, scIBD database (34), EMBL-EBI Single Cell Atlas (26) and HCA (35). We introduced two IBD single-cell webpage plug-in units originating from Mount Sinai Crohn’s single-cell data (36) and PanglaoDB’s UC single-cell data to display quick, responsive and interactive IBD single-cell data (37) (Figure 3). The web tool can provide data interaction based on cells and genes, allowing researchers to obtain a brief IBD single-cell feature and detailed gene and cell information from IBD single-cell data.

### Other resources section

Other IBD resources are also important for IBD researchers to successfully run their programs (Figure 3). First, IBD information from major IBD organizations, including the International Organization for the Study of IBD (IOIBD, https://ioibd.org), ECCO (https://www.ecco-ibd.eu) and the American Crohn’s & Colitis Foundation (CCF, https://www.crohnscolitisfoundation.org) is professional and valuable for IBD researchers. Second, IBD scoring tools such as IBD Clinical Scores Calculators (https://www.igIBDcores.it/it), IBD Biologics Precision (https://www.premedibd.com) and UC-Cancer Risk Estimator (http://www.uc-care.uk) may help IBD researchers effectively manage their volunteers. Third, important IBD single-item databases, including the UK National Institute for Health and Care Research (NIHR) IBD Bioresource (https://www.ibdbioresource.nihr.ac.uk), the IBD Genetics Consortium (https://www.ibdgenetics.org), IBDMD (https://ibdmdb.org), IBD Microbial Products Database (http://huttenhower.sph.harvard.edu/metawibele), the 1000 IBD project (https://1000ibd.org) and the IBD SCP (https://singlecell.broadinstitute.org) may greatly enrich our IBD-integrated resources and supplement the limitations. Other important biological research resources, such as the GMrepo database, EMBL-EBI Expression Atlas, Human Metabolome Database, GeneCards database, Protein Atlas and UCSC Genome database, were also sorted in our portal, which will undoubtedly benefit and facilitate IBD researchers’ programs. The homepage is the IBD brief description and the portal summary. The lab page introduces the director’s background, publications, cooperations and all lab members.

## Discussion

IBD, as heterogeneous complex diseases, involve complex interactions between genes, the environment, microbiota, immunity and potentially other factors (4–6). During the past few decades, a wealth of high-quality studies have been published to understand the physiopathology of IBD, but they can be incomprehensive and conflicting. Although several databases have been developed, such as the UCDB, IBDDB, GMrepo, IBDMDB and other IBD databases, they only provide information concerning one specific aspect. An integrated IBD resources portal still lacks; it wastes researchers’ time and delays the research project in implementing task jumping and comprehensive knowledge acquisition based on diversified needs. An integrated database of IBD is necessary to better understand the mechanism of IBD. We compiled the first IBD-integrated database with comprehensive resources, including SNPs, genes, transcriptomics, microbiota, metabolomics, single cells and other resources.

We queried GWAS and immunochip studies from the literature and sorted over 300 unique risk SNPs associated with IBD, primarily from four large-sample and high-quality studies. Some genetic variants proved to be associated with specific disease behaviors, such as NOD2 gene mutations in fibrostenosing CD (38) and variations in genes involved in maintaining the integrity and function of the intestinal barrier in UC (39). We provided the SNPs and candidate genes for these four studies separately due to different populations and criteria, and we used an UpSet diagram to present shared and unique SNP in these four studies. However, most genetic variants have a relatively minor effect on disease risk (11), and up to 80–90% of GWAS-identified loci are restricted to noncoding variations that exert their pathogenic effects via gene expression modulation (40). We also curated a highly reliable list of genes associated with IBD, providing the gene symbol, gene full name, enterz_id, associated phenotypes, experimental evidence, sample species, regulation and the study from which this result was derived. Integrating gene and SNP data may assist users in determining the likelihood that a given IBD-related gene is causative and in understanding the biological pathways crucial for IBD.

Regarding microbiota, IBD patients display reduced microbial diversity, decreased relative abundance of Firmicutes and an expansion of Proteobacteria and Bacteroidetes (14–16). Dysbiosis and relapse-related microbiomes have been identified (41), and the effects of many specific bacterial strains on IBD have been demonstrated *in vivo* (42, 43). Notably, most studies have utilized a cross-sectional design, with samples collected at a single time point. Additionally, substantial inter-individual variability in the gut microbiota composition, particularly at the subphylum level, makes it challenging to interpret meaningful connections, even in large, diverse subject groups (44). Accordingly, we presented a brief curated gut dysbiosis map of IBD derived from the GMrepo database and sorted 23 149 taxonomic records from three primary independent microbiome databases (GMrepo, gutMega and gutMDisorder).

Microbial metabolic activity is important in resisting intestinal inflammation because dietary components not digested by the small intestine enter the colon and are converted into numerous metabolites by different microbial species (45). Metabolites, byproducts of host–microbiota co-metabolomics, regulate the pathophysiology of IBD and may hold promise as a treatment for this condition. Metabolomics has been widely used to better understand host–microbe interactions by examining intestinal microbiota metabolomics and host–microbe co-metabolomicss (46). Numerous investigations have detected changes in metabolite levels in the stool, gut mucosa or serum of patients with IBD relative to controls using targeted or untargeted metabolomics. The major altered metabolites in IBD include fatty acids, amino acids and derivatives and secondary bile acids (47). We compiled 24 primary large-scale studies involving IBD metabolic profiles. We designed a table containing the title, author, IBD phenotype, sample source, sample size, population and study design of each study.

ScRNA-seq, a novel high-dimensional single-cell profiling technique, has recently been applied to samples from patients with IBD and controls, empowering unprecedented insights into refining cell-type signatures, uncovering cellular heterogeneity and suggesting new cell types (18). The number of studies using scRNA-seq is smaller than that involving the preceding sections, but the quantity and complexity of scRNA-seq data are enormous.

In the single-cell section, we listed five primary scRNA-seq data storage platforms (GEO, SCP, scIBD database, EMBL-EBI Single Cell Atlas and HCA). We introduced two scRNA-seq webpage plug-in units from Mount Sinai Crohn’s single-cell data and PanglaoDB’s UC single-cell data. This section enables researchers to interactively acquire single-cell features of IBD. Self-driven IBD single-cell data exploration can be achieved using SCP, and metadata analysis of IBD can also be performed in the scIBD database. The IBDIRP website is a task transfer station that helps researchers achieve diverse data exploration requirements.

In the past decade, large-scale omics data unraveling the pathogenesis of IBD have been rapidly generated, and several databases have been developed. Other resource sections of our platform displayed representative resources concerning IBD professional organizations, IBD management tools, IBD-specific items databases and IBD biological research resources. Our IBDIRP has several limitations and challenges. First, although we manually selected high-quality and large-scale study results, data quality from different sources may be heterogeneous. Second, our search strategy may exclude some studies that should be included, and we plan to remedy this shortcoming in future updates by incorporating other sources. The IBDIRP will be open to the feedback and suggestions from our users and is continuously improving and expanding the database based on the evolving needs of researchers.

## Conclusion

The IBDIRP website is the first integrated resource for global IBD researchers. The cross mapping, interaction and overlapping intersection based on the multidimensional data we provide can better support the IBD researcher’s programs Meanwhile, the various metadata on this website can serve as validation data for researchers to their discovery data in their own cohort. In the near future, the analysis of multi-omics data based on artificial intelligence will greatly reduce the heterogeneity and the batch effect of data on this website, providing explanations for core IBD pathophysiological processes. Notably, users can obtain integrated IBD knowledge and help their future research by reinforcing the IBD multidimensional impression. Researchers can visit the IBDIRP at www.ibdirp.com.

## Data Availability

SNP data are available in the publications (7, 8, 30–32). Gene data can be found on the IBDDB website (https://www.cbrc.kaust.edu.sa/ibd/). IBD transcriptome data are available in the GEO database (https://www.ncbi.nlm.nih.gov/geo). IBD microbiota data can be obtained from GMrepo (https://gmrepo.humangut.info), gutMega (http://gutmega.omicsbio.info) and gutMDisorder (http://bio-annotation.cn/gutMDisorder). IBD metabolomics data are accessible through curated publications in the section. IBD single-cell data can be found on data storage platforms such as GEO (https://www.ncbi.nlm.nih.gov/geo), SCP (https://singlecell.broadinstitute.org), scIBD database (http://scibd.cn/), EMBL-EBI Single Cell Atlas (https://www.ebi.ac.uk/gxa/sc/home) and HCA (https://data.humancellatlas.org/). There are two IBD single-cell plug-in units available: Mount Sinai Crohn’s single-cell data (https://scdissector.org/martin/) and PanglaoDB’s UC single-cell data (https://panglaodb.se/index.html). Other resources include IOIBD (https://ioibd.org), ECCO (https://www.ecco-ibd.eu), CCF (https://www.crohnscolitisfoundation.org), IBD Clinical Scores Calculators (https://www.igibdscores.it/it), IBD Biologics Precision (https://www.premedibd.com), UC-Cancer Risk Estimator (http://www.uc-care.uk), the UK NIHR IBD Bioresource (https://www.ibdbioresource.nihr.ac.uk), the IBD Genetics Consortium (https://www.ibdgenetics.org), the IBDMD (https://ibdmdb.org), the IBD Microbial Products Database (http://huttenhower.sph. harvard.edu/metawibele), the 1000 IBD project (https://1000ibd.org) and the IBD SCP (https://singlecell.broadinstitute.org). Other important research resources such as the GMrepo database, the EMBL-EBI Expression Atlas, the Human Metabolome Database, The GeneCards database, The Protein Atlas and the UCSC Genome database can be accessed through link buttons in the section.

## References

[1] Flynn S. and Eisenstein S. (2019) Inflammatory bowel disease presentation and diagnosis. *Surg. Clin. North Am*., 99, 1051–1062. 31676047 10.1016/j.suc.2019.08.001

[2] Kaplan G.G. and Ng S.C. (2016) Globalisation of inflammatory bowel disease: perspectives from the evolution of inflammatory bowel disease in the UK and China. *Lancet Gastroenterol. Hepatol*., 1, 307–316. 28404201 10.1016/S2468-1253(16)30077-2

[3] Bernstein C.N. (2015) Treatment of IBD: where we are and where we are going. *Am. J. Gastroenterol*., 110, 114–126. 25488896 10.1038/ajg.2014.357

[4] Ananthakrishnan A.N. , Bernstein C.N., Iliopoulos D. et al. (2018) Environmental triggers in IBD: a review of progress and evidence. *Nat. Rev. Gastroenterol. Hepatol*., 15, 39–49. 29018271 10.1038/nrgastro.2017.136

[5] de Souza H.S.P. , Fiocchi C. and Iliopoulos D. (2017) The IBD interactome: an integrated view of aetiology, pathogenesis and therapy. *Nat. Rev. Gastroenterol. Hepatol*., 14, 739–749. 28831186 10.1038/nrgastro.2017.110

[6] Chang J.T. , Longo D.L. and Longo D.L. (2020) Pathophysiology of inflammatory bowel diseases. *N. Engl. J. Med*., 383, 2652–2664. 33382932 10.1056/NEJMra2002697

[7] Liu J.Z. , van Sommeren S., Huang H. et al. (2015) Association analyses identify 38 susceptibility loci for inflammatory bowel disease and highlight shared genetic risk across populations. *Nat. Genet*., 47, 979–986. 26192919 10.1038/ng.3359 PMC4881818

[8] de Lange K.M. , Moutsianas L., Lee J.C. et al. (2017) Genome-wide association study implicates immune activation of multiple integrin genes in inflammatory bowel disease. *Nat. Genet*., 49, 256–261. 28067908 10.1038/ng.3760 PMC5289481

[9] Momozawa Y. , Dmitrieva J., Theatre E. et al. (2018) IBD risk loci are enriched in multigenic regulatory modules encompassing putative causative genes. *Nat. Commun*., 9, 2427. 10.1038/s41467-018-04365-8 PMC6013502 29930244

[10] Gettler K. , Levantovsky R., Moscati A. et al. (2021) Common and rare variant prediction and penetrance of IBD in a large, multi-ethnic, health system-based biobank cohort. *Gastroenterology*, 160, 1546–1557. 33359885 10.1053/j.gastro.2020.12.034 PMC8237248

[11] Chu H. , Khosravi A., Kusumawardhani I.P. et al. (2016) Gene-microbiota interactions contribute to the pathogenesis of inflammatory bowel disease. *Science*, 352, 1116–1120. 27230380 10.1126/science.aad9948 PMC4996125

[12] Zuo T. , Kamm M.A., Colombel J.-F. et al. (2018) Urbanization and the gut microbiota in health and inflammatory bowel disease. *Nat. Rev. Gastroenterol. Hepatol*., 15, 440–452. 29670252 10.1038/s41575-018-0003-z

[13] Human Microbiome Project Consortium . (2012) Structure, function and diversity of the healthy human microbiome. *Nature*, 486, 207–214. 22699609 10.1038/nature11234 PMC3564958

[14] Gevers D. , Kugathasan S., Denson L.A. et al. (2014) The treatment-naive microbiome in new-onset Crohn’s disease. *Cell Host Microbe*, 15, 382–392. 24629344 10.1016/j.chom.2014.02.005 PMC4059512

[15] Machiels K. , Joossens M., Sabino J. et al. (2014) A decrease of the butyrate-producing species Roseburia hominis and Faecalibacterium prausnitzii defines dysbiosis in patients with ulcerative colitis. *Gut*, 63, 1275–1283. 24021287 10.1136/gutjnl-2013-304833

[16] Nishino K. , Nishida A., Inoue R. et al. (2018) Analysis of endoscopic brush samples identified mucosa-associated dysbiosis in inflammatory bowel disease. *J. Gastroenterol*., 53, 95–106. 28852861 10.1007/s00535-017-1384-4

[17] Ahmad M.S. , Krishnan S., Ramakrishna B.S. et al. (2000) Butyrate and glucose metabolomics by colonocytes in experimental colitis in mice. *Gut*, 46, 493–499. 10716678 10.1136/gut.46.4.493 PMC1727901

[18] Hwang B. , Lee J.H. and Bang D. (2018) Single-cell RNA sequencing technologies and bioinformatics pipelines. *Exp. Mol. Med*., 50, 1–14. 10.1038/s12276-018-0071-8 PMC6082860 30089861

[19] Gedela S. (2011) Integration, warehousing, and analysis strategies of Omics data. *Methods Mol. Biol*., 719, 399–414. 21370094 10.1007/978-1-61779-027-0_18

[20] Shen J. , Mao A.P., Zhu M.M. et al. (2015) Ulcerative colitis database: an integrated database and toolkit for gene function and medication involved in ulcerative colitis. *Inflamm. Bowel. Dis*., 21, 1872–1882. 26199991 10.1097/MIB.0000000000000411

[21] Khan F. , Radovanovic A., Gojobori T. et al. (2021) IBDDB: a manually curated and text-mining-enhanced database of genes involved in inflammatory bowel disease. *Database*, 2021, baab022. 10.1093/database/baab022 PMC8086236 33929018

[22] Lloyd-Price J. , Arze C., Ananthakrishnan A.N. et al. (2019) Multi-omics of the gut microbial ecosystem in inflammatory bowel diseases. *Nature*, 569, 655–662. 31142855 10.1038/s41586-019-1237-9 PMC6650278

[23] Stark R. , Grzelak M. and Hadfield J. (2019) RNA sequencing: the teenage years. *Nat. Rev. Genet*., 20, 631–656. 31341269 10.1038/s41576-019-0150-2

[24] Barrett T. , Wilhite S.E., Ledoux P. et al. (2013) NCBI GEO: archive for functional genomics data sets—update. *Nucleic Acids Res*., 41, D991–D9995. 23193258 10.1093/nar/gks1193 PMC3531084

[25] CNCB-NGDC Members and Partners (2022) Database resources of the National Genomics Data Center, China National Center for Bioinformation in 2022. *Nucleic Acids Res*., 50, D27–D38. 34718731 10.1093/nar/gkab951 PMC8728233

[26] Cantelli G. , Cochrane G., Brooksbank C. et al. (2021) The European Bioinformatics Institute: empowering cooperation in response to a global health crisis. *Nucleic Acids Res*., 49, D29–D37. 33245775 10.1093/nar/gkaa1077 PMC7778996

[27] Wu S. , Sun C., Li Y. et al. (2020) GMrepo: a database of curated and consistently annotated human gut metagenomes. *Nucleic Acids Res*., 48, D545–D553. 31504765 10.1093/nar/gkz764 PMC6943048

[28] Zhang Q. , Yu K., Li S. et al. (2021) gutMEGA: a database of the human gut MEtaGenome Atlas. *Brief. Bioinform*., 22, bbaa082. 10.1093/bib/bbaa082 32496513

[29] Zhang X. , Fu T., Zhuang H. et al. (2020) gutMDisorder: a comprehensive database for dysbiosis of the gut microbiota in disorders and interventions. *Nucleic Acids Res*., 48, D554–D560. 31584099 10.1093/nar/gkz843 PMC6943049

[30] Huang H. , Fang M., Jostins L. et al. (2017) Fine-mapping inflammatory bowel disease loci to single-variant resolution. *Nature*, 547, 173–178. 28658209 10.1038/nature22969 PMC5511510

[31] Jostins L. , Ripke S., Weersma R.K. et al. (2012) Host-microbe interactions have shaped the genetic architecture of inflammatory bowel disease. *Nature*, 491, 119–124. 23128233 10.1038/nature11582 PMC3491803

[32] Park S.C. and Jeen Y.T. (2019) Genetic studies of inflammatory bowel disease-focusing on Asian patients. *Cells*, 8, 404. 10.3390/cells8050404 PMC6563043 31052430

[33] Liu Z. , Liu R., Gao H. et al. (2023) Genetic architecture of the inflammatory bowel diseases across East Asian and European ancestries. *Nat. Genet*., 55, 796–806. 37156999 10.1038/s41588-023-01384-0 PMC10290755

[34] Nie H. , Lin P., Zhang Y. et al. (2023) Single-cell meta-analysis of inflammatory bowel disease with scIBD. *Nat. Comput. Sci*., 3, 522–531. 38177426 10.1038/s43588-023-00464-9

[35] Hon -C.-C. , Shin J.W., Carninci P. et al. (2018) The Human Cell Atlas: technical approaches and challenges. *Brief. Funct. Genom*., 17, 283–294. 10.1093/bfgp/elx029 PMC6063304 29092000

[36] Martin J.C. , Chang C., Boschetti G. et al. (2019) Single-cell analysis of Crohn’s disease lesions identifies a pathogenic cellular module associated with resistance to anti-TNF therapy. *Cell*, 178, 1493–1508.e20. 31474370 10.1016/j.cell.2019.08.008 PMC7060942

[37] Franzén O. , Gan L.-M. and Björkegren J.L.M. (2019) PanglaoDB: a web server for exploration of mouse and human single-cell RNA sequencing data. *Database*, 2019, baz046. 10.1093/database/baz046 PMC6450036 30951143

[38] Abreu M.T. , Taylor K.D., Lin Y.C. et al. (2002) Mutations in NOD2 are associated with fibrostenosing disease in patients with Crohn’s disease. *Gastroenterology*, 123, 679–688. 12198692 10.1053/gast.2002.35393

[39] Brant S.R. , Okou D.T., Simpson C.L. et al. (2017) Genome-wide association study identifies African-specific susceptibility loci in African Americans with inflammatory bowel disease. *Gastroenterology*, 152, 206–217.e2. 27693347 10.1053/j.gastro.2016.09.032 PMC5164948

[40] McGovern D.P. , Kugathasan S. and Cho J.H. (2015) Genetics of inflammatory bowel diseases. *Gastroenterology*, 149, 1163–1176.e2. 26255561 10.1053/j.gastro.2015.08.001 PMC4915781

[41] Serrano-Gomez G. , Mayorga L., Oyarzun I. et al. (2021) Dysbiosis and relapse-related microbiome in inflammatory bowel disease: a shotgun metagenomic approach. *Comput. Struct. Biotechnol. J*., 19, 6481–6489. 34938418 10.1016/j.csbj.2021.11.037 PMC8665270

[42] ZH P.A.M. , Shi Q., Xiao X. et al. (2022) Inflammatory bowel disease-associated gut commensals degrade components of the extracellular matrix. *mBio*, 13, e0220122. 10.1128/mbio.02201-22 PMC9765649 36445085

[43] Shen Z. , Luo W., Tan B. et al. (2022) Roseburia intestinalis stimulates TLR5-dependent intestinal immunity against Crohn’s disease. *EBioMedicine*, 85, 104285. 10.1016/j.ebiom.2022.104285 PMC9526137 36182776

[44] Lee M. and Chang E.B. (2021) Inflammatory bowel diseases (IBD) and the microbiome—searching the crime scene for clues. *Gastroenterology*, 160, 524–537. 33253681 10.1053/j.gastro.2020.09.056 PMC8098834

[45] CD E.P. , Geypens B., Hiele M. et al. (1999) Amount and fate of egg protein escaping assimilation in the small intestine of humans. *Am. J. Physiol*., 277, G935–G943. 10564098 10.1152/ajpgi.1999.277.5.G935

[46] Ursell L.K. , Haiser H.J., Van Treuren W. et al. (2014) The intestinal metabolome: an intersection between microbiota and host. *Gastroenterology*, 146, 1470–1476. 24631493 10.1053/j.gastro.2014.03.001 PMC4102302

[47] Li M. , Yang L., Mu C. et al. (2022) Gut microbial metabolome in inflammatory bowel disease: from association to therapeutic perspectives. *Comput. Struct. Biotechnol. J*., 20, 2402–2414. 35664229 10.1016/j.csbj.2022.03.038 PMC9125655

